# Supplementary material for: Association between the saphenous vein diameter and venous reflux on computed tomography venography in patients with varicose veins
Source: PLoS One. 2022 Feb 15;17(2):e0263513. doi: 10.1371/journal.pone.0263513 (PMC8846520; doi:10.1371/journal.pone.0263513)
Supplement: Attachment — Submitted filename: Letterstoeditorsandreviewer0701.docx [file pone.0263513.s002.docx]

**Letters to Editors & Reviewers**

Dear Editor & Reviewers.

First of all, thank-you very much for your interest in my article.

As you have recommended me, I revised my article.

The details are followings,

**Reviewer #1:**

*1. Abstract, page 2, line 27: How does CTV identify reflux? I understand measuring diameter, but it does not measure reflux.*

I have corrected what you mentioned.

This study investigates the relationship between the diameter measured by CT and the reflux confirmed in the US, and it has been modified accordingly.

*2. Introduction, page 4, line 73: CTV as a roadmap for therapy seems like overkill to me. The saphenous vein is a straight vein and any anatomic variants are easily seen with ultrasound.*

As you mentioned, it is also known that the US can identify anatomy. However, we focus on studies that obtain information about overall anatomy and variations, past surgical performance, and so on through CT venography.

*3. Introduction, page 4, line 77: As above, I do not understand what the authors mean by CTV-proven reflux. CTV images are static and do not demonstrate reflux.*

This study is a study to investigate the relationship between the diameter measured on CT and the reflux confirmed on the US, and the mentioned parts have been corrected.

*4. Methods, page 5, line 110: Was the first duplex ultrasound on these patients performed intraoperatively? Did patients undergo preoperative duplex ultrasound to evaluate for reflux?*

We measure reflux in some patients during outpatient visits and perform it in all patients in the operating room. Therefore, ultrasound findings measured in the operating room were used as evidence.

*5. Methods, page 5, line 120: Were any adjunctive procedures, e.g., phlebectomy, sclerotherapy, performed, or just ablation.*

We did not perform sclerotherpy as a treatment option. In a small number of cases, phlebectomy was performed on the thin branch, but the core and main treatment method was ablation.

*6. Results, page 7, line 140: Was ablation performed in all patients? What was done when the intraoperative duplex did not show reflux?*

When we decided to do surgery, it was based on the dilatation of the saphenous vein through CTV, but we also performed the surgery based on the patient's symptoms and preferences.Our center performed EVLA as the basic surgical method, so ablation was performed on all patients.

There were some patients who did not show reflux because the operation was performed if the patient complained of discomfort and strongly wanted to operate even if the dilation was not severe.

*7. Results, page 7, lines 150 and 155: What were the p values for the diameter differences? They are not listed in the text and in the tables it just states <0.05.*

Each p-value was accurately corrected and recorded in the results and table.

*8. Discussion, page 9, line 185: How does preoperative CTV help to prevent intraoperative nerve injury?*

They focused on the location of saphenopopliteal junction(SPJ). SPJ morphology and the relationship between SSV and gastrocnemical vein and neural topography were important for correct removal of reflux mechanism and prevention of complication. They concluded that complete mapping of the venous networking, providing anatomical as well as hemodynamical data, was important for making decisions and surgical achievement..

I added the above to the mentioned section.

*9. Discussion, page 9, line 190: The authors cite one paper advocating high ligation in conjunction with ablation, but there is an abundance of literature in which ablation is done alone without ligation.*

As you commented, EVLA without ligation is preferred. However, we could not ignore the potential risks such as early recanalization of treated saphenous vein, development of DVT after procedure , particularly in which patients with severe varicose veins. To minimize these risks, we performed saphenofemoral junction ligation.

*10. Discussion, page 9, line 196: What do the authors mean when they state “the GSV measures 4mm (or<3mm) in diameter”? This is not clear.*

We based on our reference group.

*11. Discussion, page 9, line 208: Since the CT findings in this study are similar to reported duplex diameters in the literature for predicting reflux, what is the advantage of CT? It seems like an extra and unnecessary test to be done routinely.*

This paragraph is intended to to mention how other papers have analyzed cutoffs showing reflux based on CT or doppler before explaining our CT based cutoff diameter.

*12. Discussion, page 11, line 238: With respect to limitations, how consistent are venous diameters in the leg in serial CT exams? Veins can dilate and contract, so may be sensitive to volume status, temperature, etc.*

What you mentioned may also be a variable, but overall, we thought that CTV was a test with less variation depending on the examiner than duplex.

**Reviewer #2:**

1. *In the title nor the abstract, there is no indication of the study design. It has to be deduced from the text itself. It is not imminently discernible from the title or abstract if that is a retrospective or prospective design. That becomes clear in the material and methods section – line 86. I would suggest including the study's design in the title or abstract (as per STROBE). It might be good to include a prespecified hypothesis (e.g., we hypothesize that a positive statistically significant correlation exists between GSV/SSV diameter and pathological reflux on DUS) in the introduction section since the objective is presented clearly (»to determine the cut-off diameter of the vein that can predict reflux based on preoperative CTV«).*

Thanks for the good suggestion. I followed your recommendation and added it to the introduction.

2. *There is no clear indication of outcomes, exposures, predictors, potential confounders, and effect modifiers in the methods section. Those become clear in the results section. I would suggest defining them earlier – in the Methods section (as per STROBE).*

Thank you for your comments. I referred to it.

3. *How was the sample size arrived at? It can also be simply stated that this is a cohort under observation, and no power calculation was done*.

As described, this study is a retroactive study based on the medical records of patients who have been operated on in this hospital. Based on the sample sizes of several references, we first identified the number of EVLA cases implemented in recent years and analyzed them.

4. *In the results section (lines 144 – 156), there is a clear statement that GSV and SSV diameter was significantly larger in both regions in patients with reflux. I would suggest reporting the actual test statistics, e.g., a statistically significant difference in saphenous vein diameter of ... mm was found between both groups (95% CI, .. to ...), t(df) = ..., p < .05. From that writeup, it can be immediately deduced that a two-tailed test was used and that it was, e.g., an independent samples t-test.*

   I think it is appropriate to describe the comparison value and the p-value.
5. *A normal vein has some amount of reflux; it is physiological reflux. I would indicate (at least once) in the text that the reflux described here is pathological reflux. What is considered pathological reflux in this study is adequately stated (line 115). I would suggest including also an exact model of the ultrasound machine, not only an indication of the probe type.*

I added the exact model of the ultrasound machine

**Reviewer #3:**

*This paper is a prospective clinical study that evaluated the contribution of CTV for evaluation of saphenous vein diameter as an adjunct for diagnosis and treatment of venous disease. The underlying premise is that duplex ultrasound, which is the gold standard for diagnosis of venous reflux, is operator dependent and may not provide the best road map in patients with aberrant anatomy/needing recurrent procedures. However, the benefit of CTV as used in this study is not clear to me and I have multiple concerns.
The patients in the study were first seen in clinic where an H&P was performed and then the patients were taken for a CTV. On the CTV, the GSV and SSV diameters were measured in 2 separate locations. It appears that a duplex ultrasound, which is the only way that venous reflux is evaluated in this study (cannot be evaluated on a single phase CT), was not performed until the patients were in the operating room. Venous duplex studies to evaluated reflux are standardly performed outpatient with no sedation as they are non-invasive procedures. It is unclear in the paper what the consent process would have been to take patients to the operating room without a study demonstrating whether or not the patient needed a procedure. Once the patients were in the operating room, only 77% of extremities were evaluated for GSV reflux and only 22% of extremities were evaluated for SSV reflux. It is not clear why 100% of extremities were not evaluated for both GSV and SSV reflux, as is standard. The treatment, which was high ligation and EVLA, was reasonable. It does not appear that the CTV findings changed the surgical management or workup of the patients; all patients underwent venous duplex and patients with reflux all obtained the same ablative procedure.*

Thank-you for your comment.

First, this study was analyzed based on the medical records of patients who underwent EVLA with high ligation with varicose veins at our hospital from 2014 to 2019. Therefore, there are limitations in data verification and analysis.

In addition, preoperative ultrasound results were not included because only a limited number of patients had records of outpatient ultrasound, and all DUS was performed in the operating room, so the findings were based on intraoperative DUS findings. This is also mentioned in method.

In addition to the CTV findings and reflux, surgery was performed according to the patient's symptoms and the patient's wishes, so patients without reflux may have been included. Rather than analyzing whether it has a major impact on the CTV treatment paradigm, we wanted to check how much the diameter in the performed CT has a relationship with reflux and whether it is meaningful as a tool to predict reflux.

*Other specific concerns:
Introduction
1. For the sentence:
“Some studies have shown that reflux diagnosed on preoperative DUS reflects an increased saphenous vein diameter”
Reflux can be found in saphenous veins of any size – not sure what the authors are trying to say with this sentence. Please reword or remove.*

This paragraph is intended to mention that although many studies have been mentioned in terms of ultrasound, there are relatively few studies related to CTV.

*Methods
1. For the sentence:
“We did not perform noncontrast CT in this study owing to concerns of medical radiation.”
You don’t need to explain why a non-contrast phase was not performed for a routine CTV.*

Thanks for the recommendation. I deleted it because I thought it would be better to delete it as you mentioned.

*2. Did all patients have normal preoperative renal function? Was this checked prior to the CTV?*

We performed the basic lab before CTV in all patients, and performed CTV after confirming that the renal function was normal.

*3. Why do all of the DUS in the OR – doesn’t this mean an unnecessary OR trip for the patients who did not have reflux? Did patients get the DUS reflux study prior to the OR?*

In addition, preoperative ultrasound results were not included because only a limited number of patients had records of outpatient ultrasound, and all DUS was performed in the operating room, so the findings were based on intraoperative DUS findings.

*4. What anesthetic was used for the operative procedures?*

We performed general anesthesia.

*5. Did CTV change the operative approach?*

We used CTV as a road map, and it did not affect the surgical method itself.

*6. In postoperative follow up, how was vein obliteration and EHIT evaluated?*

It was optionally performed according to the patient's symptoms at the outpatient visit after surgery, but it was not performed as a routine procedure.

*Results
1. How many patients had prior venous procedures? If so, what approach was used? Did this change what you saw on CT in terms of vein size?*

Patients with a history of ipsilateral surgery were excluded from this study.

There were 23 cases of patients who received treatment for varicose veins on the other side.

*2. Why was reflux only measured in 77% of extremities for GSVs and 22% of extremities for SSV? Conventionally, we evaluate for both in all patients when we are evaluating venous disease.*

We performed doppler US in the operating room for all the parts to be operated on. What you mentioned is the ratio of GSV to SSV among all enrolled patients.

*3. Were the CTV diameters compared to the duplex US diameters?*

Some patients had records, we lacked a record of measuring diameter with DUS.

I’ll waiting your final acceptance mail.

Thank-you

Kind Regards.

***Corresponding Author:**

Ji Yoon Choi , MD,PhD.

Division of Transplantation and Vascular surgery, Department of Surgery, Hanyang University Medical Center, Seoul, Korea

E-mail : [skytrio6@gmail.com](mailto:skytrio6@gmail.com)

Telephone: 82-10-3374-5262
