# Supplementary material for: Association between the saphenous vein diameter and venous reflux on computed tomography venography in patients with varicose veins
Source: PLoS One. 2022 Feb 15;17(2):e0263513. doi: 10.1371/journal.pone.0263513 (PMC8846520; doi:10.1371/journal.pone.0263513)
Supplement: Attachment — Submitted filename: Letterstoeditorsandreviewer0715.docx [file pone.0263513.s003.docx]

**Letters to Editors & Reviewers**

Dear Editor & Reviewers.

First of all, I really appreciate for your interest and advice in my article.

As you have recommended me, I revised my article.

The details are followings,

**Reviewer #2: The authors addressed my concerns from 1 - 3 and 5 adequately.**

**Regarding comment No. 4, I cannot concur with the authors that the comparison value and p-value suffice. It is not good practice to state just the abovementioned values; p-value alone cannot tell readers the strength or size of an effect, change, or relationship. It should be avoided to report just the p values. It is recommended to provide the test statistics (t, F, U, etc.), correlation or regression coefficient (if applicable), or measure the effect size (https://www.editage.com/insights/the-correct-way-to-report-p-values). The above recommendations can also be found in most statistical textbooks - but with much more extended and elaborate explanations.**

**I strongly suggest reporting the actual test statistics, e.g., a statistically significant difference in saphenous vein diameter of ... mm was found between both groups (95% CI, .. to ...), t(df) =..., p < .05. From that writeup, it can be immediately deduced that a two-tailed test was used and that it was, e.g., an independent samples t-test.**

Thank-you for your comment.

As you suggested, I reviewed several statistical references.

The contents you mentioned were added to the result section and corrected, and the p-value discription of the abstract and table was also changed.

I’ll waiting your final acceptance mail.

Thank-you

Kind Regards.

***Corresponding Author:**

Ji Yoon Choi , MD,PhD.

Division of Transplantation and Vascular surgery, Department of Surgery, Hanyang University Medical Center, Seoul, Korea

E-mail : [skytrio6@gmail.com](mailto:skytrio6@gmail.com)

Telephone: 82-10-3374-5262
