# Supplementary material for: Association between the saphenous vein diameter and venous reflux on computed tomography venography in patients with varicose veins
Source: PLoS One. 2022 Feb 15;17(2):e0263513. doi: 10.1371/journal.pone.0263513 (PMC8846520; doi:10.1371/journal.pone.0263513)
Supplement: Attachment — Submitted filename: Letterstoeditorsandreviewer0803.docx [file pone.0263513.s004.docx]

**Letters to Editors & Reviewers**

Dear Editor & Reviewers.

First of all, I really appreciate for your interest and advice in my article.

Journal Requirements:

Please review your reference list to ensure that it is complete and correct. If you have cited papers that have been retracted, please include the rationale for doing so in the manuscript text, or remove these references and replace them with relevant current references. Any changes to the reference list should be mentioned in the rebuttal letter that accompanies your revised manuscript. If you need to cite a retracted article, indicate the article’s retracted status in the References list and also include a citation and full reference for the retraction notice.

I reviewed all references and there were no retracted articles.

I really thank-you for your concern.

I’ll waiting your final acceptance mail.

Thank-you

Kind Regards.

***Corresponding Author:**

Ji Yoon Choi , MD,PhD.

Division of Transplantation and Vascular surgery, Department of Surgery, Hanyang University Medical Center, Seoul, Korea

E-mail : [skytrio6@gmail.com](mailto:skytrio6@gmail.com)

Telephone: 82-10-3374-5262
