# Supplementary material for: Association between the saphenous vein diameter and venous reflux on computed tomography venography in patients with varicose veins
Source: PLoS One. 2022 Feb 15;17(2):e0263513. doi: 10.1371/journal.pone.0263513 (PMC8846520; doi:10.1371/journal.pone.0263513)
Supplement: Attachment — Submitted filename: Letterstoeditorsandreviewer0930.docx [file pone.0263513.s005.docx]

**Letters to Editors & Reviewers**

Dear Editor & Reviewers.

First of all, thank-you very much for your interest in my article.

As you have recommended me, I revised my article.

The details are followings,

***Comments to the Author***

***6. Review Comments to the Author***

***Reviewer #2: I congratulate the authors on their excellent work with the research. With the last revision, the results are reported almost accordingly to standard. I would suggest that they correct the write-up of the reported values of the test statistic (in their case, independent samples t-test or Student's t-test). The df, usually written in parentheses after t, represents the degrees of freedom; df = N-2, number of all cases in the group - 2). So in their case, it should be written for GSV; t(163) ...***

***And for SSV, it should be t(46) ... That would be the usual way to write it.***

***E.g., from the text:***

***The GSV diameter was significantly larger in both regions in patients with reflux (95% CI, -3.34 to -1.95), t(163)=-7.49, p< .05 and (95% CI, -3.49 to -2.44), t(163)=-11.96, p< .05.***

***I am sending the exact same comment as with the previous review. In the submitted text degrees of freedom are still reported as t(df) and not as the actual value. (Explanation is above - including an actual example from the text.) If it is not corrected it simply looks a bit odd. Please do correct it before publishing.***

Thank-you for your comment.

It was my mistake that I didn't correct it exactly as you said.

As you suggested, I corrected the contents..

***Reviewer #5: Thank you for your paper dealing with the association between SV diameter and venous reflux. Here are my comments:***

Thank-you for your overall comment.

Some of our related limitations and reasons are described below.

1. ***I have a serious ethical problem with your study design and your practice: which is the reason to perform an additional CT scan in a patient with varicose veins and to expose him or her to radiation? Looking at the range of the age, you have performed a completely unnecessary CT scan in patients at the age of 29. Why?***

Thank-you for your comment

Your comments are also related to the limitations of this study. As described, this study is a retroactive study based on the medical records of patients who have been operated on in this hospital.

And, we initially tried to rule out whether their symptoms or dilatation of saphenous veins are due to other structural causes (DVT or vascular abnormality, etc) through CTV when patients first visited the outpatient clinic. If there was no structural abnormality mentioned above as a result of the CTV, it was considered to be due to venous insufficiency. We also obtain information about overall anatomy and variations, past surgical performance, and so on through CTV. We performed the basic lab before CTV in all patients, and performed CTV after confirming that the renal function was normal.

2. ***You are mentioning that there is an increasing number of hospitals performing preop CTV. Firstly, I miss any references in this statement in your discussion. Secondly, which is the rationale behind this practice? And if you are taking so important informations from the CTV, which it will be extremely difficult to convince me, why not MRV?***

While we were looking for a reference, we became interested in the papers that mention the utility of CTV (Reference No.7,9,15). And we mentioned this part at the beginning of the introduction and discussion.

We performed the surgery according to the patient's symptoms and the patient's wishes, in addition to the CTV findings and reflux. As mentioned above, we performed CTV to rule out other diseases, then we used CTV as a road map, and it did not affect the surgical method itself. So, we wanted to check how much the diameter in the performed CT has a relationship with reflux and whether it is meaningful as a tool to predict reflux. rather than analyzing whether it has a major impact on the CTV treatment paradigm.

And we did not consider carry out MRV because the biggest disadvantage of MRV is that it is expensive.

***3. Although it is not important for the outcome of the study, why are you performing high ligation and EVLA and not only EVLA closely to the junction?***

Thank-you for your concern.

I know that many center prefer EVLA without ligation is as you mentioned. However, we could not ignore the potential risks such as early recanalization of treated saphenous vein, development of DVT after procedure , particularly in which patients with severe varicose veins. We searched the literature for surgical methods that can minimize recurrence, and the above methods were considered. To minimize the recurrence rate, we started saphenofemoral junction ligation.

***4. Finally, the reflux of a vein is diagnosed in a standing position. During the CTV the patient is lying on a bed. How can you associate diameters and reflux having the patient in different positions?***

We measure reflux in some patients during outpatient visits and perform it in all patients in the operating room. Therefore, ultrasound findings measured in the operating room were used as evidence. Preoperative ultrasound results were not included because only a limited number of patients had records of outpatient ultrasound, and all DUS was performed in the operating room, so the findings were based on intraoperative DUS findings. This is also mentioned in method.

I’ll waiting your final acceptance mail.

Thank-you

Kind Regards.

***Corresponding Author:**

Ji Yoon Choi , MD,PhD.

Division of Transplantation and Vascular surgery, Department of Surgery, Hanyang University Medical Center, Seoul, Korea

E-mail : [skytrio6@gmail.com](mailto:skytrio6@gmail.com)

Telephone: 82-10-3374-5262
