# Supplementary material for: Association between the saphenous vein diameter and venous reflux on computed tomography venography in patients with varicose veins
Source: PLoS One. 2022 Feb 15;17(2):e0263513. doi: 10.1371/journal.pone.0263513 (PMC8846520; doi:10.1371/journal.pone.0263513)
Supplement: Attachment — Submitted filename: Letterstoeditorsandreviewer1130.docx [file pone.0263513.s006.docx]

**Letters to Editors & Reviewers**

Dear Editor & Reviewers.

First of all, thank-you very much for your interest in my article.

I appreciate your interest and agree with some of the comments. And I would like to give you a supplementary explanation of what you pointed out.

The details are followings,

***6. Review Comments to the Author

Please use the space provided to explain your answers to the questions above. You may also include additional comments for the author, including concerns about dual publication, research ethics, or***

***Reviewer #5: I still cannot accept your response about the need for preoperative CT in young patients with varicose veins. It is not acceptable to perform CT in young patients with venous insufficiency just to see the anatomy, previous operations etc. These are no indications to justify exposure to radiation. If a patient has a arteriovenous or venovenous malformation is of course indicated, but none of your patients had such a problem. Finally, your argumentation regarding my final comment about the measurement of the vein diameter in the CT, where the patient is lying on the bed is too poor.***

There has been a change in the composition of the vascular surgeon during the period included in the study.

Previous vascular surgeons, since the 2010s, at our hospital, doppler US selectively, CT venography have been performed for several years for patients visiting outpatients with varicose veins. Recently, after becoming two vascular surgeons including myself, Doppler US is mainly performed first. This study referred to studies on the relationship between diameter and reflux after Doppler US, and studies related to CT venography and varicose veins. And, we started to compare the meaning of CT venography performed at our center. As you said, there were no cases of arteriovenous malformations in enrolled patients, but it was helpful to check the overall structure and other diseases before surgery, the shape of the recurrent varicose vein, etc., and to refer to it as a roadmap during surgery. However, even if the evaluation of renal function has been performed in consideration of the risk of contrast agents, I fully agree with the risks and limitations of radiation exposure that you mentioned. Therefore, we are in a state of control to some extent regarding the use of CTV as a diagnostic method.

I will explain the part mentioned about how to measure Diameter. As mentioned, it is common to measure reflux in a standing state. However, as previously mentioned, when referring to the medical records, the presence or absence of reflux was not evaluated on the outpatient basis in all patients, and all patients were evaluated for the presence or absence of reflux in the operating room with reverse Trendelenburg position. Therefore, we used reflux findings confirmed in the operating room as a comparison item. Since CTV is performed while lying down on a bed, I agree with your opinion that there is inevitably a difference in diameter depending on posture. At first, the analysis of the diameter of the Doppler US measured in the operating room was also considered, but there were also missing data. Therefore, the diameter of CTV in all patients was analyzed. It was measured by changing from the Supine state to the reverse Trendelenburg position, but I thought that the difference in this posture could be the cause of the vias, so I added this to the limitation.

Overall, as you said, there are limitations to our data, but we tried to use it as much as possible to analyze and find meaning.

We look forward to your understanding and good news.

Thank-you

Kind Regards.

***Corresponding Author:**

Ji Yoon Choi , MD,PhD.

Division of Transplantation and Vascular surgery, Department of Surgery, Hanyang University Medical Center, Seoul, Korea

E-mail : [skytrio6@gmail.com](mailto:skytrio6@gmail.com)

Telephone: 82-10-3374-5262
