# Supplementary material for: Association between the saphenous vein diameter and venous reflux on computed tomography venography in patients with varicose veins
Source: PLoS One. 2022 Feb 15;17(2):e0263513. doi: 10.1371/journal.pone.0263513 (PMC8846520; doi:10.1371/journal.pone.0263513)
Supplement: Attachment — Submitted filename: Letterstoeditorsandreviewer220112.docx [file pone.0263513.s007.docx]

**Letters to Editors & Reviewers**

Dear Editor & Reviewers.

First of all, I really appreciate for your interest and advice in my article.

**Journal Requirements:**

**Please review your reference list to ensure that it is complete and correct. If you have cited papers that have been retracted, please include the rationale for doing so in the manuscript text, or remove these references and replace them with relevant current references. Any changes to the reference list should be mentioned in the rebuttal letter that accompanies your revised manuscript. If you need to cite a retracted article, indicate the article’s retracted status in the References list and also include a citation and full reference for the retraction notice.**

I reviewed all references and there were no retracted articles.

I really thank-you for your concern.

**6. Review Comments to the Author**

**Please use the space provided to explain your answers to the questions above. You may also include additional comments for the author, including concerns about dual publication, research ethics, or publication ethics. (Please upload your review as an attachment if it exceeds 20,000 characters)**

**Reviewer #2: I read the comments of other reviewers. I do have to concur that doing CTV for chronic venous insufficiency without further possible indication is a highly dubious practice - it is also outside of all recommendations that I am aware of. The ethical committee that I am a member of would never grant agreement for such a study in a prospective manner. However, as you explained, you were doing retrospective (medical record) research. The CTV was not of your primary indication, and as far as I can understand, the Ethical committee agreed for such a retrospective review - I can imagine that prospective design would not be granted. With the acknowledgment that this is a retrospective design, my concerns about ethical issues are not acute (also with an acknowledgment that the Ethical committee decided on that beforehand). If this were a prospective design study - my recommendation would be to reject it due to ethical issues.

With the methodology of measuring the saphenous vein diameter - to me, it is interesting finding that there was such a big difference between refluxing and non-refluxing veins - even in the supine position (as measured per CTV). Since you did the Student t-test, it might be useful to report Cohen's d point estimate that would indicate the effect size. In my view, these findings are worth following up with US study, perhaps comparison between standing diameter and supine diameter difference compared to pathological reflux (present/absent). It might be that relative difference between standing and supine diameter will be bigger with refluxing as compared to non-refluxing veins (or vice-versa)?**

Thank-you for your recommendation.

When checking the raw data of this study, the number of US findings confirmed in outpatient clinic and operating rooms is small, and in particular, the number of diameters measured in the standing state is small. Therefore, I think it will be difficult to analyze the part you mentioned with the current data. However, it would be good to analyze the difference in diameter according to posture through prospective research through ultrasound .

Thank you for a good idea.

**Reviewer #5: Thank you for your response. Now it makes more sense and I hope that this is the real story behind. However, you have to admit all this information about the old and the new policy regarding CT venography in young patients with varicose veins and to make it crystal clear in your limitations, that this is not the way to go for preoperative scanning. Otherwise, I am sorry but you will never receive green light from me.**

Thanks for your understanding and further comment.

The limitations you mentioned are additionally mentioned at the end of the previous limitation.

In addition, it was added to the conclusion section that it should be performed according to accurate indications and guidelines.

I’ll waiting your final acceptance mail.

Thank-you

Kind Regards.

***Corresponding Author:**

Ji Yoon Choi , MD,PhD.

Division of Transplantation and Vascular surgery, Department of Surgery, Hanyang University Medical Center, Seoul, Korea

E-mail : [skytrio6@gmail.com](mailto:skytrio6@gmail.com)

Telephone: 82-10-3374-5262
